# Incidence and outcomes of critical illness in Indigenous peoples: a systematic review and meta-analysis

**DOI:** 10.1186/s13054-023-04570-y

**Published:** 2023-07-13

**Authors:** Samantha L. Bowker, Kienan Williams, Auriele Volk, Leonard Auger, Alika Lafontaine, Paige Dumont, Aireen Wingert, Amanda Davis, Liza Bialy, Erica Wright, Richard T. Oster, Sean M. Bagshaw

**Affiliations:** 1 Critical Care Strategic Clinical Network™, Alberta Health Services, 2-124 Clinical Science Building, 8440-112 Street NW, Edmonton, AB T6G 2B7 Canada; 2 Department of Critical Care Medicine, Faculty of Medicine and Dentistry, University of Alberta, and Alberta Health Services, 2-124E Clinical Science Building, 8440-112 Street NW, Edmonton, AB T6G 2B7 Canada; 3 Indigenous Wellness Core, Alberta Health Services, 10301 Southport Lane SW, Calgary, AB T2W 1S7 Canada; 4 Indigenous Medical and Dental Students Association, Faculty of Medicine and Dentistry, University of Alberta, Katz Group Centre for Pharmacy and Health Research, 1-002, Edmonton, AB T6G 2E1 Canada; 5 Indigenous Peoples and Critical Care in Alberta Advisory Committee, Faculty of Medicine and Dentistry, University of Alberta, 2-124 Clinical Science Building, 8440-112 Street NW, Edmonton, AB T6G 2B7 Canada; 6 Alberta Research Centre for Health Evidence, University of Alberta, Room 4-496A, Edmonton Clinic Health Academic, 11405 – 87 Avenue, Edmonton, AB T6G 1C9 Canada

**Keywords:** Indigenous peoples, Critical illness, Critical care, Health outcomes, Epidemiology, Systematic review

## Abstract

**Background:**

Indigenous Peoples experience health inequities and racism across the continuum of health services. We performed a systematic review and meta-analysis of the incidence and outcomes of critical illness among Indigenous Peoples.

**Methods:**

We searched Ovid MEDLINE/PubMed, Ovid EMBASE, Google Scholar, and Cochrane Central Register of Controlled Trials (inception to October 2022). Observational studies, case series of > 100 patients, clinical trial arms, and grey literature reports of Indigenous adults were eligible. We assessed risk of bias using the Newcastle–Ottawa Scale and appraised research quality from an Indigenous perspective using the Aboriginal and Torres Strait Islander Quality Assessment Tool. ICU mortality, ICU length of stay, and invasive mechanical ventilation (IMV) were compared using risk ratios and mean difference (MD) for dichotomous and continuous outcomes, respectively. ICU admission was synthesized descriptively.

**Results:**

Fifteen studies (Australia and/or New Zealand [n = 12] and Canada [n = 3]) were included. Risk of bias was low in 10 studies and moderate in 5, and included studies had minimal incorporation of Indigenous perspectives or consultation. There was no difference in ICU mortality between Indigenous and non-Indigenous (RR 1.14, 95%CI 0.98 to 1.34, I^2^ = 87%). We observed a shorter ICU length of stay among Indigenous (MD − 0.25; 95%CI, − 0.49 to − 0.00; I^2^ = 95%) and a higher use for IMV among non-Indigenous (RR 1.10; 95%CI, 1.06 to 1.15; I^2^ = 81%).

**Conclusion:**

Research on Indigenous Peoples experience with critical care is poorly characterized and has rarely included Indigenous perspectives. ICU mortality between Indigenous and non-Indigenous populations was similar, while there was a shorter ICU length of stay and less mechanical ventilation use among Indigenous patients.

*Systematic Review Registration* PROSPERO CRD42021254661; Registered: 12 June, 2021.

**Supplementary Information:**

The online version contains supplementary material available at 10.1186/s13054-023-04570-y.

## Background

Indigenous Peoples are distinct legal, social and cultural groups that share collective ancestral ties to the lands and natural resources where they live, occupy or from which they have been displaced [1]. The Canadian *Constitution Act* formally recognizes three groups of Indigenous Peoples: First Nations, Métis, and Inuit, all of which are distinct peoples with unique histories, languages, cultural practices and spiritual beliefs [2]. Health inequities for Indigenous Peoples around the globe are well documented and are rooted within the ongoing and multi-generational impacts of colonization and racism, which need to be contextualized within the historical, political, social, and economic conditions that have influenced of Indigenous health [3–6]. These inequities span across the healthcare continuum from birth to death and are exacerbated by disparities in the social determinants of health and structural racism endemic within healthcare systems [7–11].

Prioritizing equity requires that we build a healthcare system that meets the unique needs of Indigenous Peoples to overcome barriers to the provision of high-quality services, to recognize and respect Indigenous leadership over their own health matters, and to create culturally safe health service environments and practices [12, 13]. A culturally safe healthcare system, inclusive of critical care and intensive care units (ICUs), is one key action to reduce health inequities experienced by Indigenous Peoples [14–17]. There has been no systematic evaluation of critical illness or critical care use among Indigenous Peoples.

Accordingly, we performed a systematic review and meta-analysis to describe the use of critical care services, including the incidence of critical illness and critical care outcomes among Indigenous Peoples, compared to non-Indigenous counterparts. The findings are informing a larger program of work, which includes co-designing transformative research with Indigenous Peoples and creating an ethical space for researchers and Indigenous community members to come together in relationship and trust [20].

## Methods

This systematic review and meta-analysis was guided by standard evidence synthesis methodology outlined in the Cochrane Handbook for Systematic Reviews of Interventions [21] and reported according to the Preferred Reporting Items in Systematic Reviews and Meta-Analyses (PRISMA) guidelines and the Meta-Analysis of Observational Studies in Epidemiology (MOOSE) guidelines for observational studies [22]. Our protocol was registered with PROSPERO International Prospective Register of Systematic Reviews (Registration number: CRD42021254661; June 12, 2021) [23].

For this review, critical illness was defined by complexity of illness, severity of organ dysfunction and risk of mortality that necessitates receipt of advanced monitoring or life support (e.g., invasive mechanical ventilation [IMV]) that can only be delivered in an ICU setting [18, 19]. We defined critical care access and/or utilization as admission to an ICU or support in a hospital location designated as an ICU for ≥ 24 h.

### Search strategy

The search strategy was developed in consultation with the Alberta Research Centre for Health Evidence (ARCHE) at the University of Alberta and conducted by an information specialist. The search strategy included the following two groups of terms (key words with similar characteristics): ‘Indigenous Peoples’ and ‘critical care’ (Additional file 1: File S1). We systematically searched the following electronic databases from inception to October 2022: Ovid MEDLINE/PubMed, Ovid EMBASE, Google Scholar, and Cochrane Central Register of Controlled Trials (Additional file 1: File S1). We limited results to human studies that were published in English. We also complemented this search by scanning potentially relevant websites for grey literature (National Collaborating Centre for Indigenous Health, First Nations Health Authority, Canadian Institutes of Health Research Institute of Indigenous Peoples’ Health, National Association of Friendship Centres, the First Nations Information Governance Centre, Métis Nation of Alberta; Australia Institute of Health and Welfare [Indigenous Health and Wellbeing]; New Zealand Ministry of Health [Manatū Hauora and Māori Health]; United States Department of Health and Human Services [Indian Health Service]). Lastly, we hand-searched bibliographies of included studies and relevant reviews for additional citations. We exported bibliographic records into EndNote X9 (Thomas Reuters, Philadelphia, PA, USA) database for screening and removal of duplicate citations.

### Eligibility criteria

Retrospective and prospective observational cohort studies, case series reporting aggregate data on > 100 patients, arms of clinical trials (e.g., usual care, control, or placebo arm), and analytical data from grey literature reports of Indigenous adults (≥ 18 years) either without critical illness (i.e., general population) or with critical illness (i.e., admitted to an ICU) were all eligible for inclusion. Descriptive studies, cross-sectional studies, case-reports, and articles that do not present original data (e.g., editorials, commentaries, narrative reviews) were excluded.

Our primary outcome measures were ICU admission and ICU mortality. For ICU admission, we only included studies with a non-Indigenous ‘general population’ or ‘hospitalized’ comparator. Studies in the general or hospitalized population that did not have a non-Indigenous comparison group were only included if they also had information on the following factors within the Indigenous population: age, sex, and illness acuity. For ICU mortality, we included studies with and without a non-Indigenous comparator. Secondary outcomes of interest included ICU length of stay, ICU re-admission, receipt IMV, duration of IMV, receipt of tracheostomy, receipt of vasoactive support, duration of vasoactive support, acute kidney injury (AKI), receipt of renal replacement therapy (RRT), duration of RRT, and quality of life.

### Study selection

**Fig. 1:**
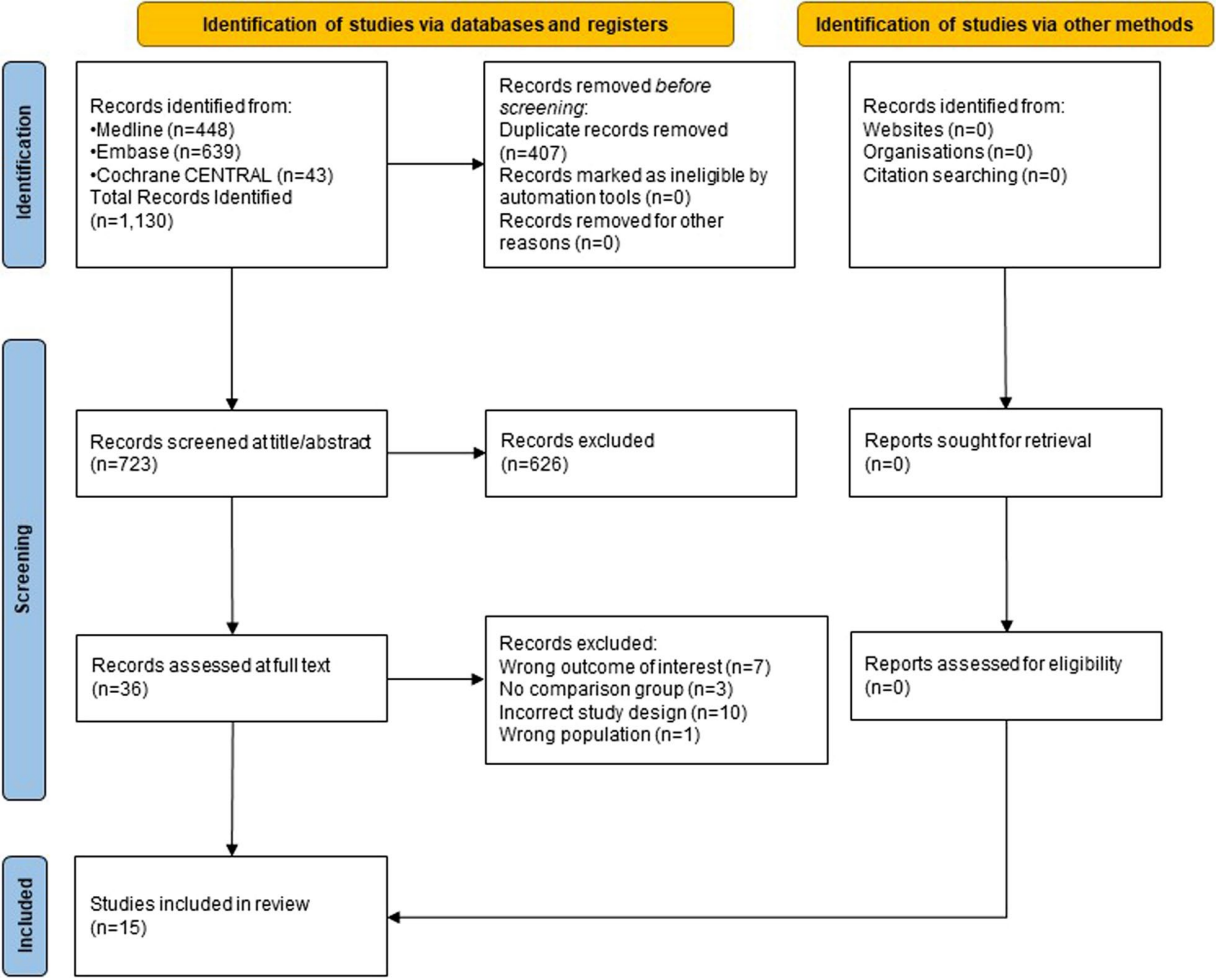
PRISMA flow diagram for identification, screening, and inclusion of studies

Both abstract title (Level 1) and full text (Level 2) reviews had pre-determined eligibility criteria. Level 1 criteria were broader than Level 2 to ensure all potentially relevant studies were captured. Two reviewers independently, and in duplicate, reviewed the titles and abstracts of the retrieved citations. Full-text articles were obtained for all titles and abstracts identified by one or both reviewers as potentially relevant. Subsequently, two reviewers independently, and in duplicate, identified full-text articles that met the inclusion criteria. Any eligibility disagreements between reviewers were resolved through discussion and a third reviewer with clinical expertise, when required (Fig. 1) [22].

### Data extraction

For each study, one reviewer extracted data into a standardized electronic form, while a second reviewer verified the extracted data. Discrepancies at any stage were resolved through discussion and referred to the third reviewer, when required.

### Risk of bias assessment of studies

Two reviewers independently assessed the risk of bias (RoB) using the Newcastle–Ottawa Scale (NOS) across the following domains: selection of the exposed and unexposed cohorts, comparability of the cohorts, and outcome ascertainment (Additional file 1: File S2) [24]. We required that groups were comparable, or controlled for age, sex/gender, and level of acuity, at a minimum. We summed scores the primary outcomes (ICU admission and ICU mortality); studies were rated as high risk of bias (< 4/9), moderate risk of bias (4–6/9), or low risk of bias (> 6/9). Any disagreement in ratings between reviewers were resolved by discussion or by consulting a third reviewer.

### Data analysis and synthesis

We classified studies first according to their risk of bias for the primary outcomes of ICU admission and ICU mortality. There was adequate clinical and methodological homogeneity to perform a meta-analysis for ICU mortality, ICU length of stay, and receipt of IMV (Review Manager version 5.4, the Nordic Cochrane Centre, the Cochrane Collaboration, Copenhagen, Denmark). We did not pool data for the outcome of ICU admission, due to substantial differences in the presentation of data across the four studies that reported this outcome. Therefore, findings for this outcome are summarized using a descriptive synthesis approach for systematic reviews [25].

Dichotomous outcomes (ICU mortality and IMV) were analyzed using risk ratio and random effects, whereas continuous outcomes (ICU length of stay) were analyzed using mean difference. Data that were reported as medians and interquartile ranges were imputed as means and converted to standard deviations, respectively. Denominators reported as number of admissions were imputed as number of people, where appropriate, to permit pooling. Heterogeneity was quantified using the I^2^ statistic and was explored using between-study subgroup analyses (i.e., by case-mix). We also performed sensitivity analyses to understand the effects of variably defined exposures and/or outcomes. For meta-analyses that included at least eight studies of varying size (i.e., ICU length of stay), we tested for small study bias by interpreting funnel plots and statistically using Egger’s test [26]. Statistical analyses were performed using Stata SE version 13.1 (Stata Corp, LP, College Station, TX).

### Confidence in cumulative evidence

Two reviewers independently assessed the certainty of evidence for the primary outcomes using the Grading of Recommendations Assessment, Development, and Evaluation (GRADE) approach [27, 28]. As the best evidence for prognostic factors originates from observational studies [29], evidence from these started at high certainty, and were rated down for concerns about risk of bias, inconsistency, indirectness, imprecision, and other concerns. Inconsistencies between reviewers were resolved by discussion or the involvement of a third reviewer, if needed. We used “probably”, “may be”, or “uncertain” to reflect level of certainty in the evidence based on GRADE of moderate, low, or very low, respectively.

### Qualitative appraisal of literature – Indigenous perspective

In addition to scientifically appraising evidence through a Western lens, we aimed to describe and evaluate the quality of research from an Indigenous perspective. We used the 14-item Aboriginal and Torres Strait Islander Quality Assessment Tool (ATSI QAT) to appraise included studies (Additional file 1: File S3) [30]. The ATSI QAT items focus on understanding, from an Indigenous perspective, whether the research responds to a community need, has community and Indigenous leadership engagement, has negotiated agreements for access to and protection of Indigenous intellectual and cultural property, provides benefits to Indigenous participants and communities, and enables Indigenous ownership of data collection and management [30]. Two reviewers independently assessed the included studies using the ATSI QAT, with each item assessed as “yes”, “partial”, “unclear”, or “no”. Any discrepancies between reviewers were resolved by discussion or the involvement of a third reviewer. We considered studies to have a higher quality, from an Indigenous perspective, if they had a higher number of “yes” assessments. We summed the assessments for the 14 ATSI QAT items for each study. We ranked the 15 studies to identify the highest and lowest quality from an Indigenous perspective.

### Ethics approval and project oversight

Ethics approval was not required for this study. We invited our Indigenous Peoples and Critical Care Advisory Committee (IPCCAC), which is comprised of Métis (n = 2) and First Nation (n = 3) individuals, to review, appraise and offer perspective to the findings of this systematic review. This exchange was facilitated through dialogue and aimed to consider alternative interpretation and incorporation of feedback from the IPCCAC. This systematic review represents a foundational project within a larger program of culturally appropriate, respective, and mutually beneficial work aimed at understanding the lived experience of Indigenous Peoples with critical care, to be co-designed with Indigenous partners.

## Results

The PRISMA flow diagram for identification, screening, and inclusion of studies is shown in Fig. 1. We retrieved 1,130 records from Medline (n = 448), Embase (n = 639) and Cochrane Central (n = 43). After removing duplicates (n = 407), we screened 723 records at title and abstract (Level 1) and 36 records at full text (Level 2), resulting in 15 studies fulfilling eligibility [31–45].

Among the 15 studies, 12 were conducted in Australia and/or New Zealand and 3 in Canada. All included studies were observational and either retrospective (n = 12) or prospective (n = 3) cohort study designs, focused on the adult populations, and included Indigenous vs. non-Indigenous groups for comparison (Table 1). Among the 15 studies, Indigenous populations were generally younger, more likely female, and were more likely to have pre-existing comorbid disease compared with non-Indigenous populations. Illness acuity scores were generally similar between populations; however, admission diagnosis of sepsis was generally higher among Indigenous compared with non-Indigenous, where applicable.

**Table 1:** Characteristics of included studies in the systematic review

Study author and year of publication	Country (no. sites)	Study design	Data source & timeframe	Patients (n)	Population and comparison group	Patient characteristics		Outcome measures	Risk of bias (NOS)¶
						Indigenous	Non-Indigenous		
Davis JS et al. 2011	Australia (1)	Prospective cohort	Hospital medical records/charts, pathology results May 2007-May 2008	1,090	Adults > 15 years hospitalized with community-onset sepsis Indigenous vs. non-Indigenous	Age: 43.2 yr Male: 43.2% DM: 31.1% CKD: 18.9% CLD: 13.2% Sepsis: 100% APA II: N/A* SOFA: N/A**	Age: 50.2 yr Male: 61.8% DM: 16.5% CKD: 4.4% CLD: 5.3% Sepsis: 100% APA II: N/A* SOFA: N/A**	ICU admission	5
Dunlop WA et al. 2020	Australia (177)	Retrospective cohort	ANZICS Core national database Jan 1, 2010 – Dec 31, 2017	23,793	Adults admitted to ICU with dialysis-dependent ESKD Indigenous vs. non-Indigenous	Age: 57.0 yr Male: 52.9% DM: 17.5% CKD: 100% CLD: 3.1% Sepsis: 19.0% ANZROD: 15	Age: 70.5 yr Male: 62.0% DM: 17.3% CKD: 100% CLD: 2.8% Sepsis: 14.2% ANZROD: 16	ICU LOS; ICU re-admission	7
Flint SM et al. 2010	Australia (1)	Prospective cohort	Hospital medical records; lab data June – August 2009	643	Adults hospitalized with influenza-like illness Indigenous vs. non-Indigenous	Age: 39 yr Male: 47.0% DM: 24.0% CKD: 20.0% CLD: 11.0% > 1 comorbid: 74.0% APA II: N/A^α^	Age: 46 yr Male: 64.0% DM: 21.0% CKD: 5.0% CLD: 5.0% > 1 comorbid: 72.0% APA II: N/A^α^	ICU admission	5
Hanson J et al. 2020	Australia (1)	Retrospective cohort	Medical records; lab data Jan 1, 2014 – Jun 30, 2017	442	Adults admitted to ICU with sepsis Indigenous vs. non-Indigenous	Age: 53 yr Male: 47.0% DM: 58.0% CKD: 39.0% CLD: 12.0% Sepsis: 100% APA II: 21.0 ANZROD: 24	Age: 65 yr Male: 57.0% DM: 23.0% CKD: 10.0% CLD: 7.0% Sepsis: 100% APA II: 20.0 ANZROD: 23	ICU mortality, IMV	6 (ICU admission); 8 (ICU mortality)
Ho KM et al. 2006	Australia (1)	Retrospective cohort	ICU Audit database Jan 1, 1993 – Dec 31, 2003	16,757	Adults admitted to ICU for elective surgery, emergency Indigenous vs. non-Indigenous	Age: 42.2 yr Male: 58.0% DM: N/A CKD: 7.0% CLD: 2.3% Sepsis: 14.5% APA II: 18.6 ANZROD: N/A	Age: 57.9 yr Male: 66.0% DM: N/A CKD: 1.0% CLD: 0.7% Sepsis: 4.0% APA II: 16.9 ANZROD: N/A	ICU mortality; ICU LOS	6
Jung JJ et al. 2017	Canada (51)	Prospective cohort	ICU-FLU electronic database Apr 16, 2009 – Apr 12, 2010	647	Adults admitted to ICU for pH1N1 Indigenous vs. non-Indigenous	Age: 40.7 yr Male: 35.8% DM: 23.5% CKD: N/A CLD: N/A ≥ 1 comorbid: 92.6% APA II: 19.9	Age: 49.0 yr Male: 49.9% DM: 26.9% CKD: N/A CLD: N/A ≥ 1 comorbid: 91.0% APA II: 21.1	ICU LOS; IMV	7
Keenan NM et al. 2019	Australia (1)	Retrospective cohort	Flinders Cardiac Surgery Research database Feb 1, 1992 – Jul 31, 2017	236	Adult undergoing redo heart valve surgery Indigenous vs. non-Indigenous	Age: 29.5 yr Male: 41.0% DM: 17.0% CKD: 18.0% CLD: N/A EuroSCORE II: 3.7	Age: 67.0 yr Male: 68.0% DM: 19.0% CKD: 12.0% CLD: N/A EuroSCORE II: 4.3	ICU LOS	7
Khan NA et al. 2008	Canada (3)	Retrospective cohort	ICU database registry Jan 1999 – Jan 2006	7,331	Adults admitted to ICU Indigenous vs. European vs. Asian	Age: 48.4 yr Male: 48.0% DM: 19.0% CKD: N/A CLD: N/A IVDU: 30.0% Sepsis: 22.0% APA II: 21.6	Age: 59.2 yr Male: 64.0% DM: 17.0% CKD: N/A CLD: N/A IVDU: 8.0% Sepsis: 12.0% APA II: 20.4	ICU mortality; ICU LOS	6
Laupland KB et al. 2006	Canada (4)	Retrospective cohort	ICU Tracer database May 1, 1999 – Apr 30, 2002	6,272	Adults admitted to ICU/CV ICU Indigenous (Status Aboriginal) vs. non-Indigenous (non-Status Aboriginal)	Age: 40.3 yr Male: 58.0% DM: N/A CKD: 5.0% CLD: N/A Sepsis: N/A APA II: 22.1	Age: 65.3 yr Male: 63.0% DM: N/A CKD: 2.0% CLD: N/A Sepsis: N/A APA II: 24.9	ICU mortality; ICU LOS; ICU admission; IMV	8 (ICU admission); 9 (ICU mortality)
Magee F et al. 2019	Australia & New Zealand (92)	Retrospective cohort	ANZICS Adult patient database Jan 1, 2010 – Dec 31, 2015	23,804	Adults > 17 years old admitted to ICU for trauma Indigenous vs. non-Indigenous	Age: 42.0 yr Male: 73.4% DM: 1.1% CKD: 1.7% CLD: 1.7% APA III: 27.0 ANZROD: 7.6	Age: 48.3 yr Male: 74.0% DM: 1.2% CKD: 0.7% CLD: 0.9% APA III: 26.1 ANZROD: 8.8	ICU LOS; ICU admission; IMV	7
Maiden MJ et al. 2020^γ^	Australia & New Zealand (183)	Retrospective cohort	ANZICS Adult patient database Jan 1, 2008 – Dec 31, 2017	16,063	Obstetric patients 15–49 years admitted to ICU Indigenous vs. non-Indigenous	Age: 31.3 yr DM: N/A CKD: N/A CLD: N/A Sepsis: 5.0% APA III: 32 ANZROD: 1.3		ICU mortality; ICU LOS; IMV	6
Mitchell WG et al. 2020	Australia (4)	Retrospective cohort	ANZICS Adult patient database Jan 1, 2007 – Dec 31, 2016	39,784	Adults admitted non-electively to ICU Indigenous vs. non-Indigenous	Age: 45.0 yr Male: 54.0% DM: 32.0% CKD: 8.5% CLD: 6.2% Sepsis: 11.0% APA II: 17.0 ANZROD: N/A	Age: 64.0 yr Male: 58.0% DM: 20.0% CKD: 2.9% CLD: 2.6% Sepsis: 9.3% APA II: 18.0 ANZROD: N/A	ICU mortality; ICU LOS; IMV	7
Reid Al et al. 2022	New Zealand (17)	Retrospective cohort	New Zealand Ministry of Health National Minimum Dataset matched to ANZICS Adult patient database July 1, 2009 – June 30, 2018	52,552	Adult patients ≥ 18 years old admitted to ICU Maori vs. European	Age: 53 yr Male: 55.6% DM: 30.2% CKD: 12.8% CLD: N/A Sepsis: 7.6% APA III: 50.6 ANZROD: 10.6	Age: 66 yr Male: 62.7% DM: 17.7% CKD: 7.9% CLD: N/A Sepsis: 4.4% APA III: 46.0 ANZROD: 9.5	ICU mortality; ICU LOS	9
Secombe PJ et al. 2019	Australia & New Zealand (148)	Retrospective cohort	ANZICS Adult patient database Jan 1, 2017 – Dec 31, 2018	246,718	Adults admitted to ICU Indigenous vs. non-Indigenous	Age: 51.1 yr Male: 51.8% DM: N/A CKD: 9.4% CLD: 2.7% > 1 comorbid: 4.5% Sepsis: 14.9% APA III: 47.0 ANZROD: 8.6	Age: 66.0 yr Male: 56.5% DM: N/A CKD: 3.0% CLD: 1.5% > 1 comorbid: 4.8% Sepsis: 9.1% APA III: 47.0 ANZROD: 8.1	ICU mortality; ICU LOS; ICU re-admission; IMV	7
Trout MI et al. 2015^β^	Australia (1)	Retrospective cohort	ANZICS Adult patient database Jan 1, 2007 – Dec 31, 2011	2,019	Adults admitted to ICU ATSI vs. non-ATSI	Age: 53.0 yr Male: 56.0% DM: 7.4% CKD: 5.2% CLD: 1.4% Sepsis: 3.2% APA III: 38 ANZROD: N/A	Age: 63.0 yr Male: 72.2% DM: 3.6% CKD: 0.8% CLD: 0.3% Sepsis: 1.4% APA III: 42 ANZROD: N/A	ICU mortality; ICU LOS; IMV	7

ICU = intensive care unit; LOS = length of stay; ANZICS = Australia and New Zealand Intensive Care Society; IMV = invasive mechanical ventilation; ATSI = Aboriginal and/or Torres Strait Islander; ESKD = end-stage kidney disease; DM = diabetes mellitus; CKD = chronic kidney disease; CLD = chronic liver disease; APA II = APACHE II score; SOFA = Sequential Organ Failure Assessment score; ANZROD = Australia New Zealand Risk of Death score; EuroSCORE II = European system for cardiac operative risk evaluation score; IVDU = intravenous drug use

^¶^Newcastle–Ottawa Scale (NOS) maximum score is 9; poor quality (< 4/9), moderate quality (4–6/9), high quality (> 6/9). The score applies to ICU admission and ICU mortality, unless otherwise denoted

*APA II score (median [IQR]) provided for cohort aggregate only (total 8 [4–13]; severe sepsis 16 [9–22]; non-severe sepsis 6 [3–10])

**SOFA score (median [IQR]) provided for cohort aggregate only (total 1 [0–3]; severe sepsis 4 [2–7]; non-severe sepsis 1 [0–2])

^α^APA II score (median [IQR]) provided for cohort aggregate only (total 16 [14–23]) among 28 patients (17%) admitted to ICU

^γ^Data available for aggregate only

^β^Proportions of Indigenous and non-Indigenous patients with selected comorbid disease and sepsis were estimated based on data available

The following primary and secondary outcomes were reported: ICU admission/incidence of critical illness (n = 4), ICU mortality (n = 9), ICU length of stay (n = 12), receipt of IMV (n = 8), and ICU re-admission (n = 2).

The RoB was considered low in 9 studies [32, 36, 37, 39, 40, 42–45] and moderate in 5 studies [31, 33, 35, 38, 41]. The study by Hanson et al. [34] was assessed as moderate RoB for ICU admission but low RoB for ICU mortality (Table 1 and Additional file 1: File S2). The reasons for demerits across studies were ascertainment (lack thereof) of Indigenous status, adequate comparability of groups, and insufficient follow-up. Among studies that reported on ICU admission, two studies each were considered at low [39, 40] and moderate [31, 33] RoB, respectively. Among studies that reported on ICU mortality, six [34, 39, 42–45] and two [35, 38] were at low and moderate RoB, respectively.

The included studies provided minimal detail in their study design, methods and results on Indigenous aspects or involvement according to the ATSI QAT (Additional file 1: File S3). All studies scored low on the ATSI QAT (no study scored either “Yes” or “Partial” on two or more of the 14 questions). Only 2 studies received a ‘Yes’ rating for describing a response to an Indigenous community need and consultation [35, 39].

### Primary outcome—ICU admission

The definition and description of ICU admission across studies was heterogeneous: number of ICU admissions rather than number of patients [31]; estimated ICU incidence with age-adjusted incidence rate ratio [33]; annual incidence of ICU admission with relative risk [39]; and estimated ICU admission with incidence ratio [40] (Additional file 1: Files S4 and S5; Table 2). Among cohorts that compared general populations or hospitalized patients, there was suggestion of increased risk of ICU admission for Indigenous when compared with non-Indigenous populations (low certainty evidence). In the study by Laupland et al., the annual incidence of critical illness among Indigenous Peoples was significantly higher (620.6 admissions per 100,000 population) compared with the general Canadian population (302.6 admissions per 100,000 population) (RR 2.1, 95% CI, 1.78 to 2.35; P < 0.0001), which was consistently observed across all age groups [39]. Three studies examined ICU admission among specific patient subgroups, including sepsis [31], pandemic H1N1 influenza A [33], and trauma-related injuries [40]. In these examples, Indigenous patients had higher incidences of ICU admission compared to non-Indigenous patients [31, 33, 40].

**Table 2:** GRADE summary of findings comparing Indigenous versus non-Indigenous for primary outcomes

**Outcome**	Comparator 1 vs. 2	Study design (no. studies); Sample size	Absolute difference (95% CI)		Effect estimate (95% CI)	Certainty of evidence^†^	Conclusion
			Comparator 2 risk	Absolute risk difference			
ICU admission	Indigenous vs non-Indigenous	Prospective (2), Retrospective (2); Sample size range 161 to 18,742,918 people	NA	NA	ICU admissions among hospital admissions for severe sepsis: 4.7 per 1,000 (95% CI 3.8 to 5.7) vs. 0.9 per 1,000, p < 0.001; ICU admissions among patients hospitalized for H1N1: 35.3 per 100,000 (95% CI 16.5 to 54.4) vs. 5.7 per 100,000 (95% CI 1.8 to 9.9); age-adjusted incidence rate ratio: 5.2 (95% CI 2.3 to 12), p > 0.05; Annual incidence of ICU admissions among general population: 620.6 per 100,000 vs. 302.6 per 100,000; RR 2.1 (95% CI 1.78 to 2.35), p < 0.0001; ICU admissions with trauma-related injuries among general population: 847 per 1,000,000 vs. 251 per 1,000,000; incidence ratio 3.37 (95% CI 3.19 to 3.57), standardized to estimates for adult population of Australia	Low^1,2^	Among general populations or hospitalized patients, there may be an increased risk in ICU admission for Indigenous compared with non-Indigenous populations
ICU mortality	Indigenous vs non-Indigenous	Retrospective cohort (8); 368,541 people in ICU	65 per 1,000	16 more per 1,000 (2 fewer to 40 more)	RR 1.14 (95% CI 0.98 to 1.34)	Low^1,3^	Among people in ICU, there may be little-to-no difference in ICU mortality between Indigenous and non-Indigenous populations

CI: confidence interval; GRADE: Grading of Recommendations Assessment, Development and Evaluation; ICU: intensive care unit; NA: not applicable; no.: number; RR: rate ratio; vs.: versus

^†^Certainty of evidence was assessed for each outcome using GRADE methodology, starting at high for prognosis evidence, and downgrading ((if any) for one or more of the domains of study limitations, inconsistency, indirectness, imprecision, and reporting/publication bias

^1^One decrement for inconsistency

^2^One decrement for reporting bias

^3^One decrement for indirectness

### Primary outcome—ICU mortality

**Fig. 2:**
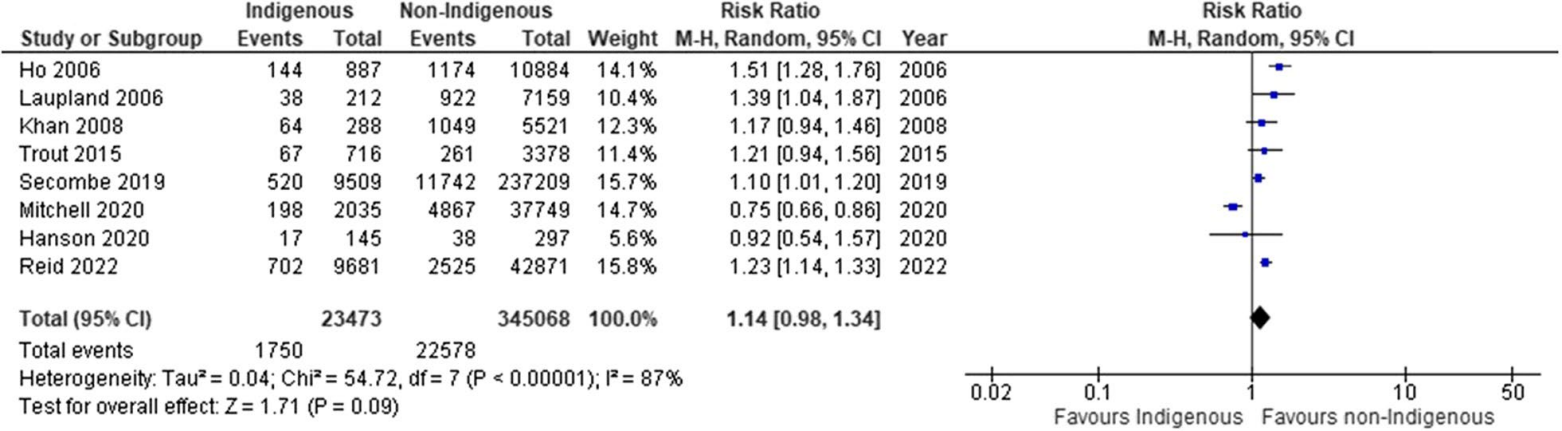
Forest plot of Indigenous versus non-Indigenous for ICU mortality: any indication

Nine studies reported on ICU mortality [34, 35, 38, 39, 41–45], of which, eight were pooled (Fig. 2; Table 2; Additional file 1: File S6A and S7A). We found no statistical difference between Indigenous and non-Indigenous populations for ICU mortality (8 studies; RR 1.14, 95% CI, 0.98 to 1.34; absolute RD 16 more per 1,000; 95% CI, 2 fewer to 40 more; I^2^ = 87%; low certainty evidence), regardless of clinical indication for admission. Two studies reported on patients admitted emergently (i.e., unplanned) or electively to ICU [35, 44]. Among emergent patients, we observed a significantly higher ICU mortality among the non-Indigenous patients (2 studies; RR 1.18, 95% CI, 1.03 to 1.35) (Additional file 1: File S6A).

The study by Maiden et al. reported on a subgroup of obstetric critically ill patients aged 15–49 years old and was not included in any of our pooled analyses [41]. In this subgroup, we found no statistically significant difference in ICU mortality between Indigenous Australian Torres Strait Islander patients in Australia (RR 1.11, 95% CI, 0.51 to 2.40) or Māori (Indigenous) patients in New Zealand (RR 1.16, 95% CI, 0.32 to 4.26), compared to non-Indigenous patients (Additional file 1: File S7A) [41].

### Secondary outcomes

**Fig. 3:**
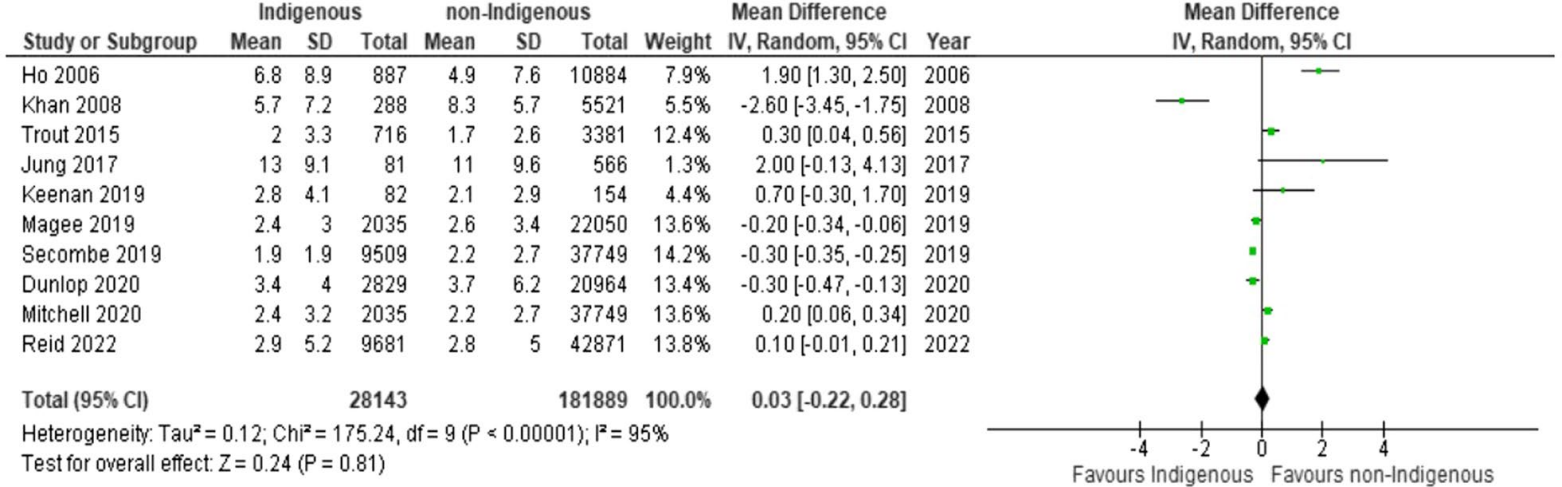
Forest plot of Indigenous versus non-Indigenous for ICU length of stay: any indication. In a sensitivity analysis, the study by Khan et al. [38] was omitted from the analysis due to being an outlier. There was no significant influence on the effect estimate after this study was omitted (MD, 0.16; 95% CI, -0.07 to 0.40, p = 0.18; I^2^ = 94%).

Twelve studies reported on ICU length of stay [32, 35–45] (Fig. 3; Table 2; Additional file 1: File S6B and 7B). Two studies were omitted from pooled analysis, due to one reporting on obstetric critically ill patients [41] and the other not reporting a measure of variance [39]. We found no significant difference between Indigenous and non-Indigenous populations for ICU length of stay (10 studies; MD 0.03, 95% CI, -0.22 to 0.28; I^2^ = 95%), regardless of clinical indication for admission (Fig. 3). We found no evidence of small study bias for ICU length of stay (Egger’s test, p = 0.19; Additional file 1: File S8).

**Fig. 4:**
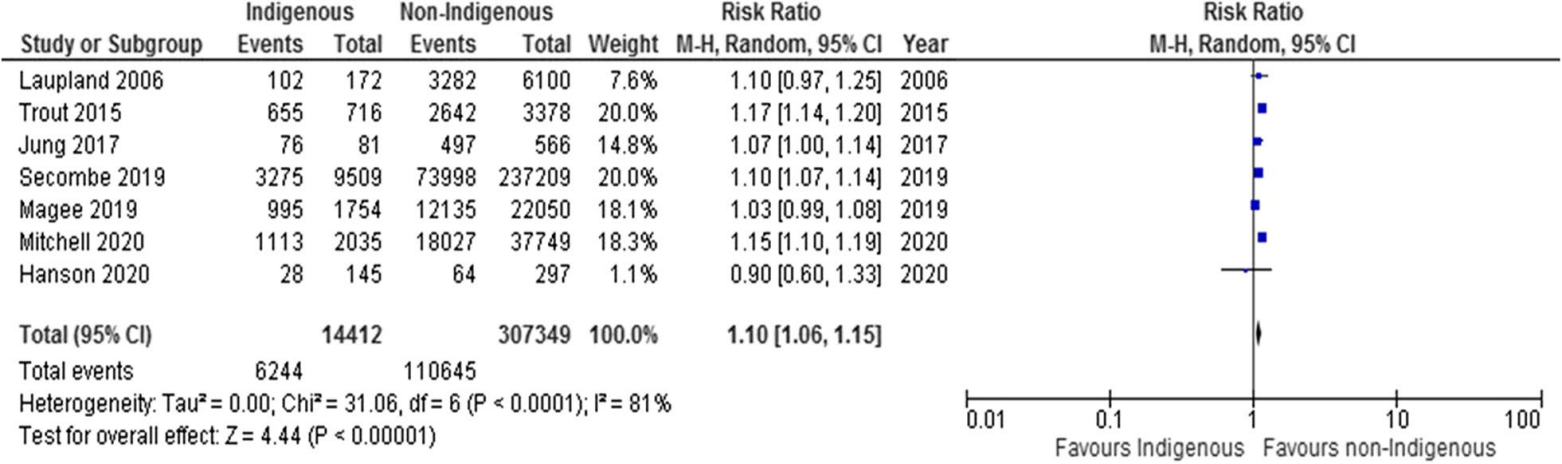
Forest plot of indigenous versus non-indigenous for IMV: any indication

Seven studies reported on receipt of IMV [34, 36, 39, 40, 42–44] (Fig. 4; Table 2). We found that non-Indigenous patients were significantly more likely to receive IMV in ICU compared to Indigenous patients (7 studies; RR 1.10, 95% CI, 1.06 to 1.15; I^2^ = 81%). This was similarly shown in sensitivity analyses of subgroups (Additional file 1: File S6C and S7C).

We were not able to perform pooled analyses on other pre-specified secondary outcomes (Additional file 1: File S4). Kidney outcomes were variably reported across four studies: RRT [34]; AKI and AKI treated with RRT, among patients undergoing redo aortic and/or mitral valve surgery [37, 43]; and AKI on day 1 [39]. Hanson et al. was the only study reporting on use of vasopressors [34]. There was no difference in vasopressors use between Indigenous and non-Indigenous patients. Two studies reported rates of ICU readmission. Secombe et al. found Indigenous patients were more likely to experience readmission to ICU in subsequent hospitalizations compared with non-Indigenous patients [43]. The study by Dunlop et al. compared Indigenous and non-Indigenous patients with kidney failure receiving chronic RRT [32]. While the reported rate of ICU admission was higher among Indigenous compared with non-Indigenous patients receiving chronic RRT, rates of ICU readmission within the index hospitalization were similar [32]. No studies reported on the receipt of tracheostomy or on quality of life among survivors.

## Discussion

### Key findings

We performed a rigorous systematic review and evidence synthesis to describe the incidence of critical illness and associated outcomes among Indigenous compared with non-Indigenous populations. Importantly, we found that Indigenous consultation, involvement, and perspectives were rarely considered in the design, methodology or interpretation of the results across these studies. Few studies compared the incidence of critical illness among Indigenous and non-Indigenous populations; however, among these, Indigenous populations were generally found to have higher rates of critical illness and receive support in ICU settings comparatively [31, 33, 39, 40]. Our evidence synthesis did not find differences in ICU mortality between Indigenous and non-Indigenous populations. While our evidence synthesis suggested that Indigenous critically ill patients had shorter stays in ICU and were less likely to receive IMV, inferences from these findings may be limited due to significant heterogeneity across studies, likely due to bias and residual confounding.

### Context with prior literature

An important consideration in systematically evaluating the published literature is to not only use established Western methodologies, but also appraise the literature through an Indigenous perspective. As such, we applied the validated ASTI QAT to qualitatively appraise the included studies through an Indigenous lens. We found that the current understanding of Indigenous Peoples experiences with critical illness and with critical care is limited and poorly characterized. Importantly as well, the existing literature has largely adopted a Western research approach. Moreover, the existing literature appears to have largely failed to seek Indigenous consultation and perspectives in the research process, has not adopted co-design or Indigenous research methodology, has not provided details on Indigenous Data Governance (such as Ownership, Control, Access, and Possession (OCAP™), has not sought an Indigenous lens for interpretation, and has not commented on whether the research output had any direct impact on Indigenous communities [30, 46].

As such, our evidence synthesis should be contextualized with prior work that has focused on Indigenous Peoples experience in other acute care settings, particularly, the emergency department (ED). A population-based study in Alberta, Canada found that First Nations Peoples were three-fold more likely to visit the ED compared with non-First Nations people, despite only comprising 4% of the total population [47]. These authors further showed that status as First Nations was associated with lower odds of receiving higher acuity triage scores across several different diagnoses and conditions upon presentation to the ED [48]. Further follow-up work has suggested that overt systemic racism and stereotyping of First Nations patients occurs in the ED [6]. Numerous barriers to care were identified, including issues related to communication, health system access (e.g., access to primary care), and the cultural safety of the ED environment. While this may partly explain the higher rate of ED utilization among First Nations Peoples, this does not account for the enduring systemic cultural or structural barriers propagating health inequity among Indigenous patients (i.e., triage acuity scores).

Similar findings have been observed in EDs in Australia and New Zealand. A systematic review found that Aboriginal and Torres Strait Islanders visited EDs twice as often as non-Indigenous Australians, with Indigenous patients also more likely to leave the ED prior to being evaluated [49]. In a cohort study from New Zealand, Māori patients were found to have worse outcomes after visiting the ED, including higher mortality and ED re-presentation, compared with non-Indigenous patients [50]. The authors concluded that these health inequities were not driven by differences in process measures (i.e., assessment and disposition times) in the ED [50]. The findings of our evidence synthesis may align with these experiences in ED settings. The observed shorter ICU stays and lower rates of receipt of IMV among Indigenous compared with non-Indigenous patients raises important questions of whether similar issues of systemic and structural racism and pedagogy exist in critical care settings.

### Strengths and limitations

First, our systematic review is strengthened by a priori publication of a protocol, inclusion of a rigorous and comprehensive peer-reviewed search strategy, and systematic evaluation of the quality and risk of bias of included studies, all using established Western methodologies [23]. Second, we applied the ASTI QAT to qualitatively appraise the included studies for an Indigenous perspective. Third, we further invited members of local Indigenous communities, who formed an Advisory Committee, to offer perspective and an Indigenous-specific lens to the co-design and to the interpretation of our findings.

Our systematic review also has limitations to consider. First, the definition of “Indigenous” and the availability of “identifiers” in health administrative varied substantially, driven by differences in legal definitions across countries and by academic discussions on culturally appropriate terminology. Indeed, the lack of suitable or validated identifiers for Indigenous Peoples in health data represents a barrier to understanding the impact of structural racism, complex post-traumatic stress disorder and inter-generational trauma on health access and outcomes [13]. Second, as shown by our analyses of the ATSI QAT scores, included studies were largely led by non-Indigenous researchers using Western methodologic approaches, which may have contributed to implicit biases that impact both the analysis and interpretation. Third, studies were appraised as being at low-to-moderate risk of bias, all studies were focused in only three high-income countries, were all observational, and showed marked heterogeneity across outcomes of interest. As such, generalizability is limited, and any inferences and interpretation should be conservative. Further, health systems and access likely differ across the included studies (Australia, New Zealand, and Canada); therefore, we have been cautious in comparing the findings from the different countries. Lastly, we acknowledge that the general population comparison groups were likely highly variable and heterogenous among studies. However, it was not within the scope of this systematic review and meta-analysis to tease out these effects.

### Implications for healthcare professionals, health policy, and future research

Our evidence synthesis would strongly imply there is a narrow and incomplete understanding of Indigenous Peoples risk of critical illness and their experiences with critical care (i.e., ICU environment). The scope and magnitude of health inequities in access to ICU support and outcomes after critical illness, if existing, remains poorly described and represents a barrier to action. These observations imply further work is urgently needed. Moreover, this work would align directly with key recommendations for health within the *Truth and Reconciliation Commission of Canada: Calls to Action* report and further honors the principles of the *United National Declaration on the Rights of Indigenous Peoples* [13, 51]. Ideally, Indigenous together with non-Indigenous researchers and their communities would partner to co-develop and co-learn to better characterize and understand Indigenous Peoples’ (i.e., patients, families, and communities) experiences and outcomes with critical care, to identify knowledge and care gaps, and to work toward ensuring culturally appropriate and safe space.

## Conclusions

Indigenous Peoples continue to experience health inequities, precipitated and driven by the legacy of colonization and inter-generational trauma. Despite this, Indigenous Peoples’ experiences with critical care is poorly documented and understood. Existing literature describing Indigenous Peoples and critical care has rarely sought Indigenous consultation, co-design, or perspective in the research process or in the interpretation of findings. Pooled analysis suggested Indigenous and non-Indigenous populations had similar rates of ICU mortality; however, Indigenous populations were found to experience shorter durations of ICU stay and lower likelihood of receipt of IMV when compared to non-Indigenous populations. Many other secondary outcomes of interest were inadequately reported. These findings reinforce the urgency of additional work, co-developed with Indigenous partners, on the experience of Indigenous Peoples with critical care, and further interpreted through a lens of Indigenous Ways of Knowing.

## Supplementary Information

**Additional file 1: File S1**. Full search strategy and search terms. **File S2**. Risk of bias assessments for included studies, using the Newcastle–Ottawa Scale. **File S3**. Summary of the Aboriginal and Torres Strait Islander Quality Assessment Tool appraisal. **File S4**. Ancillary analysis of outcomes not included in the main manuscript. **File S5**. GRADE evidence profile comparing Indigenous vs. non-Indigenous populations for the primary outcomes. **File S6.** Forest plots for of Indigenous vs. non-Indigenous for ICU mortality (Figure s6A), ICU Length of Stay (Figure s6B), and IMV (Figure s6C): subgroup analysis by indication. **File S7**. Forest plots of Indigenous vs. non-Indigenous populations among an obstetric critically ill population for: ICU mortality (Figure s7A), ICU LOS (Figure s7B), and IMV (Figure s7C). **File S8**. Egger’s Funnel plot for small study bias.

## Data Availability

All data generated or analyzed during this study are included in this published article (and its supplementary information files).

## Abbreviations

ATSI QAT: Aboriginal and Torres Strait Islander Quality Assessment Tool
AKI: Acute kidney injury
ARCHE: Alberta Research Centre for Health Evidence
CI: Confidence interval
ED: Emergency department
GRADE: Grading of Recommendations Assessment, Development, and Evaluation
HIC: High income countries
ICU: Intensive care unit
IMV: Invasive mechanical ventilation
IPCCAC: Indigenous Peoples and Critical Care Advisory Committee
MD: Mean difference
MOOSE: Meta-Analysis of Observational Studies in Epidemiology
NOS: Newcastle-Ottawa Scale
OCAP™: Ownership, Control, Access, and Possession
PRISMA: Preferred Reporting Items in Systematic Reviews and Meta-Analyses
RoB: Risk of bias
RR: Relative risk/risk ratio
RRT: Renal replacement therapy
