# Supplementary material for: Incidence and outcomes of critical illness in Indigenous peoples: a systematic review and meta-analysis
Source: Crit Care. 2023 Jul 13;27:285. doi: 10.1186/s13054-023-04570-y (PMC10339531; doi:10.1186/s13054-023-04570-y)

**Supplementary Files.**

**Supplementary File 1.** Full search strategy and search terms.

**Database: Ovid MEDLINE(R) ALL <1946 to May 03, 2021>**

Search Strategy: Indigenous-Critical Care MEDLINE

--------------------------------------------------------------------------------

1 exp American Native Continental Ancestry Group/ (21889)

2 exp Indigenous Peoples/ (4819)

3 Health Services, Indigenous/ (3463)

4 Northern Territory/ (1501)

5 Arctic Regions/ (6798)

6 Nunavut/ (345)

7 Oceanic Ancestry Group/ (10744)

8 aborigin*.ti,ab,kf. (10374)

9 ((American or Northamerican) adj1 Indian*).ti,ab,kf. (7215)

10 amerindian*.ti,ab,kf. (2034)

11 (First adj1 (Nation or Nations or People)).ti,ab,kf. (2197)

12 indigenous*.ti,ab,kf. (35424)

13 (eskimo* or inuit*).ti,ab,kf. (3375)

14 maori*.ti,ab,kf. (3669)

15 metis*.ti,ab,kf. (445)

16 (Native* adj1 (American* or Alaska* or Australia* or Canadian* or Northamerican*

or Hawaiian*)).ti,ab,kf. (10628)

17 Nunavik*.ti,ab,kf. (250)

18 Nunavut*.ti,ab,kf. (567)

19 Torres Strait Islander*.ti,ab,kf. (1828)

20 or/1-19 (84520) [Indigenous Population Terms]

21 Critical Care/ (54606)

22 intensive care units/ (58790)

23 ((intensive or critical) adj (care or medicine)).ti,ab,kf. (181739)

24 (ICU or ICUs).ti,ab,kf. (66609)

25 intensivist*.ti,ab,kf. (3826)

26 Critical Illness/ (31668)

27 critical* ill*.ti,ab,kf. (55820)

28 Multiple Organ Failure/ (11272)

29 (multi* organ adj (disfunction* or dis function* or dysfunction* or dys function* or

failure*)).ti,ab,kf. (15806)

30 (multi* system adj (disfunction* or dis function* or dysfunction* or dys function* or

failure*)).ti,ab,kf. (292)

31 or/21-30 (279966) [Critical Care Terms]

32 20 and 31 (342)

33 limit 32 to english language (334)

***************************

**Database: Embase <1974 to 2021 May 03>**

Search Strategy: Indigenous-Critical Care Embase

--------------------------------------------------------------------------------

1 exp indigenous people/ (28355)

2 indigenous health care/ (962)

3 northern territory/ (462)

4 Arctic/ (8060)

5 nunavut/ (179)

6 exp oceanic ancestry group/ (7545)

7 exp eskimo-aleut people/ (2927)

8 exp malayo-polynesian people/ (4433)

9 mestizo/ or metis/ (810)

10 aborigin*.ti,ab,kw. (12957)

11 ((american or northamerican) adj1 Indian*).ti,ab,kw. (8740)

12 amerindian*.ti,ab,kw. (2592)

13 (First adj1 (Nation or Nations or People)).ti,ab,kw. (2917)

14 indigenous*.ti,ab,kw. (42766)

15 (eskimo* or inuit*).ti,ab,kw. (3412)

16 maori*.ti,ab,kw. (4579)

17 metis*.ti,ab,kw. (598)

18 (Native* adj1 (American* or Alaska* or Australia* or Canadian* or

Northamerican*)).ti,ab,kw. (12718)

19 Nunavik*.ti,ab,kw. (277)

20 Nunavut*.ti,ab,kw. (627)

21 Torres Strait Islander*.ti,ab,kw. (2401)

22 or/1-21 (100577)

23 intensive care/ or intensive care nursing/ (132971)

24 intensive care unit/ (171573)

25 ((intensive or critical) adj (care or medicine)).ti,ab,kw. (275595)

26 (ICU or ICUs).ti,ab,kw. (135255)

27 intensivist*.ti,ab,kw. (7113)

28 critical illness/ (31703)

29 critical* ill*.ti,ab,kw. (85817)

30 multiple organ failure/ (41983)

31 (multi* organ adj (disfunction* or dis function* or dysfunction* or dys function* or

failure*)).ti,ab,kw. (25883)

32 (multi* system adj (disfunction* or dis function* or dysfunction* or dys function* or

failure*)).ti,ab,kw. (454)

33 or/23-32 (498333)

34 22 and 33 (750)

35 limit 34 to conference abstracts (272)

36 34 not 35 (478)

37 limit 36 to english language (465)

***************************

**Cochrane Library on Wiley**

Date Run: 05/05/2021 18:52:15

ID Search Hits

#1 [mh "American Native Continental Ancestry Group"] (296)

#2 [mh ^"Health Services, Indigenous"] (42)

#3 [mh ^"Medically Underserved Area"] (123)

#4 [mh ^"Northern Territory"] (22)

#5 [mh ^Nunavut] (2)

#6 [mh ^"Oceanic Ancestry Group"] (173)

#7 [mh "Indigenous Peoples"] (49)

#8 aborigin*:ti,ab,kw (339)

#9 ((American or Northamerican) near/1 Indian*):ti,ab,kw (567)

#10 Eskimo*:ti,ab,kw (34)

#11 (First near/1 (Nation or Nations or People*)):ti,ab,kw (187)

#12 Indigenous*:ti,ab,kw (925)

#13 Inuit*:ti,ab,kw (61)

#14 Maori*:ti,ab,kw (237)

#15 Metis*:ti,ab,kw (40)

#16 (Native* near/1 (American* or Alaska* or Australia* or Canadian* or

Northamerican* or Hawaiian*)):ti,ab,kw (488)

#17 Nunavik*:ti,ab,kw (1)

#18 Nunavut*:ti,ab,kw (6)

#19 Torres Strait Islander*:ti,ab,kw (87)

#20 (or #1-#19) (2512)

#21 [mh ^"Critical Care"] (1762)

#22 [mh ^"intensive care units"] (2369)

#23 ((intensive or critical) near/1 (care or medicine)):ti,ab,kw (25821)

#24 (ICU or ICUs):ti,ab,kw (13854)

#25 intensivist*:ti,ab,kw (307)

#26 [mh "critical illness"] (2353)

#27 critical* ill*:ti,ab,kw (11231)

#28 [mh "Multiple Organ Failure"] (417)

#29 (multi* organ near/1 (disfunction* or dis function* or dysfunction* or dys function*

or failure*)):ti,ab,kw (3587)

#30 (multi* system near/1 (disfunction* or dis function* or dysfunction* or dys

function* or failure*)):ti,ab,kw (540)

#31 (or #21-#30) (39473)

#32 #20 AND #31 in Trials (34)

***************************

**Supplementary File 2.** Risk of bias assessments for included studies, using the Newcastle-Ottawa Scale.

| **Study** | **Selection** | | | | **Comparability** | **Outcome** | | | **Total***  **a) ICU admission**  **b) ICU mortality** |
| --- | --- | --- | --- | --- | --- | --- | --- | --- | --- |
|  | **Representative of exposed cohort** | **Selection of non-exposed cohort** | **Ascertainment of exposure** | **Demonstration outcome not present at start of study** | **Comparability of subjects**  **a) ICU admission**  **b) ICU mortality** | **Outcome assessment** | **Adequate follow-up for outcomes** | **Adequate follow-up of cohorts** |  |
| Davis 2011 | 1 | 1 | 0 | 1 | a) 0  b) NA | 1 | 1 | 0 | a) 5  b) 5 |
| Dunlop 2020 | 1 | 1 | 1 | 1 | a) 0  b) NA | 1 | 1 | 1 | a) 7  b) 7 |
| Flint 2010 | 1 | 1 | 0 | 1 | a) 0  b) NA | 1 | 1 | 0 | a) 5  b) 5 |
| Hanson 2020 | 1 | 1 | 0 | 1 | 1. NA 2. 2 | 1 | 1 | 1 | a) 6  b) 8 |
| Ho 2006 | 1 | 1 | 0 | 1 | a) NA  b) 0 | 1 | 1 | 1 | a) 6  b) 6 |
| Jung 2017 | 1 | 1 | 1 | 1 | a) NA  b) NA | 1 | 1 | 1 | a) 7  b) 7 |
| Keenan 2019 | 1 | 1 | 1 | 1 | a) NA  b) NA | 1 | 1 | 1 | a) 7  b) 7 |
| Khan 2008 | 1 | 1 | 0 | 1 | a) NA  b) 0 | 1 | 1 | 1 | a) 6  b) 6 |
| Laupland 2006 | 1 | 1 | 1 | 1 | a) 1  b) 2 | 1 | 1 | 1 | a) 8  b) 9 |
| Magee 2019 | 1 | 1 | 1 | 1 | a) 0  b) NA | 1 | 1 | 1 | a) 7  b) 7 |
| Maiden 2020 | 1 | 1 | 1 | 1 | 1. NA 2. NA | 1 | 1 | 0 | a) 6  b) 6 |
| Mitchell 2020 | 1 | 1 | 1 | 1 | 1. NA 2. 0 | 1 | 1 | 1 | a) 7  b) 7 |
| Reid 2022 | 1 | 1 | 1 | 1 | a) 2  b) 2 | 1 | 1 | 1 | a) 9  b) 9 |
| Secombe 2019 | 1 | 1 | 1 | 1 | 1. NA 2. 0 | 1 | 1 | 1 | a) 7  b) 7 |
| Trout 2015 | 1 | 1 | 1 | 1 | a) NA  b) 0 | 1 | 1 | 1 | a) 7  b) 7 |

*Risk of bias was assessed using the Newcastle-Ottawa Scale (NOS): maximum score is 9; poor quality (<4/9), moderate quality (4-6/9), high quality (>6/9).

ICU: intensive care unit; NA: not applicable

**Supplementary File 3:** Summary of the Aboriginal and Torres Strait Islander Quality Assessment Tool appraisal.

| **Reference** | **Q.1** | **Q.2** | **Q.3** | **Q.4** | **Q.5** | **Q.6** | **Q.7** | **Q.8** | **Q.9** | **Q.10** | **Q.11** | **Q.12** | **Q.13** | **Q.14** |
| --- | --- | --- | --- | --- | --- | --- | --- | --- | --- | --- | --- | --- | --- | --- |
| Davis 2011 | X | X | X | X | X | X | X | X | X | X | **P** | X | X | X |
| Dunlop 2020 | X | X | X | X | X | X | X | X | X | X | **P** | X | X | X |
| Flint 2010 | X | X | X | X | X | X | X | X | X | X | X | X | X | X |
| Hanson 2020 | X | X | X | X | X | X | X | X | X | X | **P** | X | X | X |
| Ho 2006 | ✔ | ✔ | X | X | X | X | X | X | X | X | X | X | X | X |
| Jung 2017 | X | **U** | X | X | X | X | X | X | X | X | X | X | X | X |
| Keenan 2019 | X | X | X | X | X | X | X | X | X | X | **P** | **P** | X | X |
| Khan 2008 | X | X | X | X | X | X | X | X | X | X | X | X | X | X |
| Laupland 2006 | ✔ | ✔ | X | **P** | X | X | X | X | **P** | X | X | X | X | X |
| Magee 2019 | X | X | X | X | X | X | X | X | X | X | X | X | X | X |
| Maiden 2020 | X | X | X | X | X | X | X | X | X | X | X | X | X | X |
| Mitchell 2020 | **P** | **P** | X | X | X | X | X | X | X | X | X | X | X | X |
| Reid 2022 | X | X | X | X | X | X | X | X | X | X | X | X | X | X |
| Secombe 2019 | X | X | **P** | X | X | X | X | X | X | X | X | X | X | X |
| Trout 2015 | X | **U** | **U** | **U** | X | X | X | X | X | X | X | X | X | X |

✔= Yes; P = Partially; X = No; U = Unclear

Aboriginal and Torres Strait Islander Quality Assessment Tool questions.

| 1. Did the research respond to a need or priority determined by the community? 2. Was community consultation and engagement appropriately inclusive? 3. Did the research have Aboriginal and Torres Strait Islander research leadership? 4. Did the research have Aboriginal and Torres Strait Islander governance? 5. Were local community protocols respected and followed? 6. Did the research negotiate agreements in regard to rights and access to Aboriginal and Torres Strait Islander peoples’ existing intellectual and cultural property? 7. Did the research negotiate agreements to protect and access to Aboriginal and Torres Strait Islander peoples’ ownership intellectual and cultural property created through the research? 8. Did Aboriginal and Torres Strait people and communities have control over the collection and management of research materials? 9. Was the research guided by an Indigenous research paradigm? 10. Does the research take a strengths-based approach, acknowledging and moving beyond practices that have harmed Aboriginal and Torres Strait Islander people in the past? 11. Did the researchers plan and translate the findings into sustainable changes in policy and/or practice? 12. Did the research benefit the participants and Aboriginal and Torres Strait Islander communities? 13. Did the research demonstrate capacity strengthening for Aboriginal and Torres Strait Islander individuals? 14. Did everyone involved in the research have opportunities to learn from each other? |
| --- |

**Supplementary File 4:** Ancillary analysis of outcomes not included in the main manuscript.

| **Study** | **Results (Indigenous vs. non-Indigenous)** |
| --- | --- |
| **ICU admission** | |
| Davis 2011 | **ICU admissions among hospital admissions for severe sepsis:**  20.1%; 4.7 per 1,000 (95% CI, 3.8 to 5.7) for Indigenous vs. 11.9%; approximately 0.9 per 1,000 for non-Indigenous; p<0.001 |
| Flint 2010 | **ICU admissions among patients hospitalized with H1N1:**  35.3 per 100,000 estimated resident population (95% CI, 16.5 to 54.4) for Indigenous vs. 5.7 per 100,000 estimated resident population (95% CI, 1.8 to 9.9) for non-Indigenous.  Age-adjusted incidence rate ratio: 5.2 (95% CI, 2.3 to 12); p>0.05 |
| Laupland 2006 | **Critical illness/ICU admission among general population:**  Annual incidence: 620.6 per 100,000 for Indigenous vs. 302.6 per 100,000 for non-Indigenous;  RR 2.1 (95% CI, 1.78 to 2.35); p<0.0001  **Admission to multisystem ICU among general population:**  Annual incidence 579.6 per 100,000 for Indigenous vs. 210.7 per 100,000 for non-Indigenous;  RR 2.75 (95% CI, 2.37 to 3.17); p<0.0001  **Admission to cardiovascular surgery ICU among general population:**  Annual incidence 41.0 per 100,000 for Indigenous vs. 91.8 per 100,000 for non-Indigenous;  RR 0.45 (95% CI 0.24 to 0.75); p<0.001 |
| Magee 2019 | **ICU admissions with trauma-related injuries among general population:**  847 admissions (among estimated 423,319 Indigenous adults) per 1,000,000 vs. 251 admissions (among estimated 18,319,599 non-Indigenous adults) per 1,000,000;  Incidence ratio 3.37 (95% CI, 3.19 to 3.57), standardized to Australian Bureau of Statistics population estimates for adult population of Australia |
| **Kidney outcomes** | |
| Hanson 2020 | **RRT among patients in ICU with sepsis:**  19/145 (13.1%) vs. 27/297 (9.1%), p=0.20 |
| Keenan 2019 | **AKI among patients undergoing redo valve surgery:**  15/82 (18.3%) vs. 17/154 (11%), p=1.0  **AKI receiving RRT among patients undergoing redo valve surgery:**  3/82 (3.7%) vs. 6/154 (3.9%), p=1.0 |
| Laupland 2006 | **AKI on day 1 among patients in ICU:**  8/172 (4.7%) vs. 138/6,100 (2.3%), p=0.06 |
| Secombe 2019 | **AKI among patients in ICU:**  671/9,509 (7.1%) vs. 10,130/237,209 (4.3%), p<0.001 |
| **Vasopressors** | |
| Hanson 2020 | **Vasopressors on admission among patients in ICU with sepsis:**  102/145 (70.3%) vs. 204/297 (68.7%), p=0.72  **Number of vasopressors required among patients in ICU with sepsis**, median (IQR):  1 (1 to 1) vs. 1 (0 to 1), p=0.89 |
| **ICU readmission** | |
| Dunlop 2020 | **>1 ICU admission among patients with RRT-dependent CKD:**  303/2,829 (10.7%) vs. 2,390/20,964 (11.4%);  OR 0.9 (95% CI 0.8 to 1.1) |
| Secombe 2019 | **ICU readmission during subsequent hospitalization among hospital survivors**:  661/8,774 (7.5%) vs. 12,257/218,782 (5.6%), p<0.001 |

CI: confidence interval; ICU: intensive care unit; OR: odds ratio; RR: relative risk; SAC: status Aboriginal Canadian; vs.: versus; RRT: renal replacement therapy; AKI: acute kidney injury; CKD: chronic kidney disease

* age-adjusted ICU admission rate per 100,000 was estimated by using PlotDigitizer (<https://automeris.io/WebPlotDigitizer/>) to extract data from the box plot.

**Supplementary File 5:** GRADE evidence profile comparing Indigenous vs. non-Indigenous populations for the primary outcomes.

| **Outcome** | **Results** | 1. **Risk of bias** | 1. **Inconsistency** | 1. **Indirectness** | 1. **Imprecision** | 1. **Reporting bias** | **Overall**  **certainty^†^** |
| --- | --- | --- | --- | --- | --- | --- | --- |
| ICU admission | 4 studies  (see ICU admission – descriptive synthesis, below) | **No concerns**  Low risk of bias  (n=2; Laupland, Magee)  Moderate risk of bias (n=2; Davis, Flint) | **Serious concerns**  No significant difference between groups (1 study, n=161)  vs.  Increased ICU admission among Indigenous population (3 studies, n>18,750,000) | **No concerns**  Studies included adults admitted to ICU for range of indications (sepsis, influenza, trauma, cardiovascular, unspecified) | **No concerns**  OIS adequate (>18,750,000 total sample) | **Serious concerns**  4 studies (n>18,750,000) reporting outcome  vs.  8 studies (n=313,934) missing outcome;  publication bias not detected | Low |
| ICU mortality among patients in ICU | 8 studies  RR 1.14 (95% CI 0.98 to 1.34) | **No concerns**  Low risk of bias (n=6; Hanson, Laupland, Mitchell, Ried Secombe, Trout)  Moderate risk of bias (n=2; Ho, Khan) | **Serious concerns**  Equal number of studies with estimates on both sides, with most converging around null, and CIs overlapping; high heterogeneity, not explained by subgroup analysis | **Serious concerns**  Studies included adults admitted to ICU, with mainly unspecified indications including emergency, non-elective admissions, elective surgery, and sepsis;  1 study reported number of admissions rather than number of patients (imputed) | **No concerns**  OIS adequate (1,750 events/ 23,473 people  vs.  22,578 events/ 345,068 people); CI includes no effect | **No concerns**  8 studies (n=368,541) reporting outcome  vs.  6 studies (n=50,213)  missing outcome;  publication bias not detected | Low |

^†^ Certainty of evidence was assessed for each outcome using GRADE methodology, starting at high for prognosis evidence, and downgrading ((if any) for one or more of the domains of study limitations, inconsistency, indirectness, imprecision, and reporting bias

**Supplementary File 6:** Forest plots for of Indigenous vs. non-Indigenous ICU mortality, ICU LOS, and MV: subgroup analysis by indication.

**eFigure 6A.** Forest plot of ICU mortality: subgroup analysis by indication.


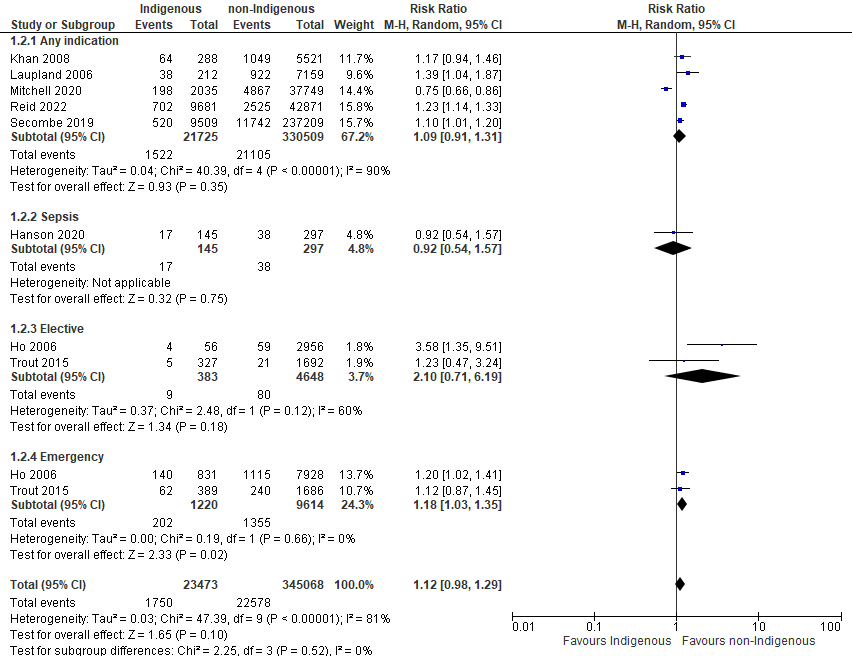


**eFigure 6B.** Forest plot of ICU length of stay: subgroup analysis by indication.


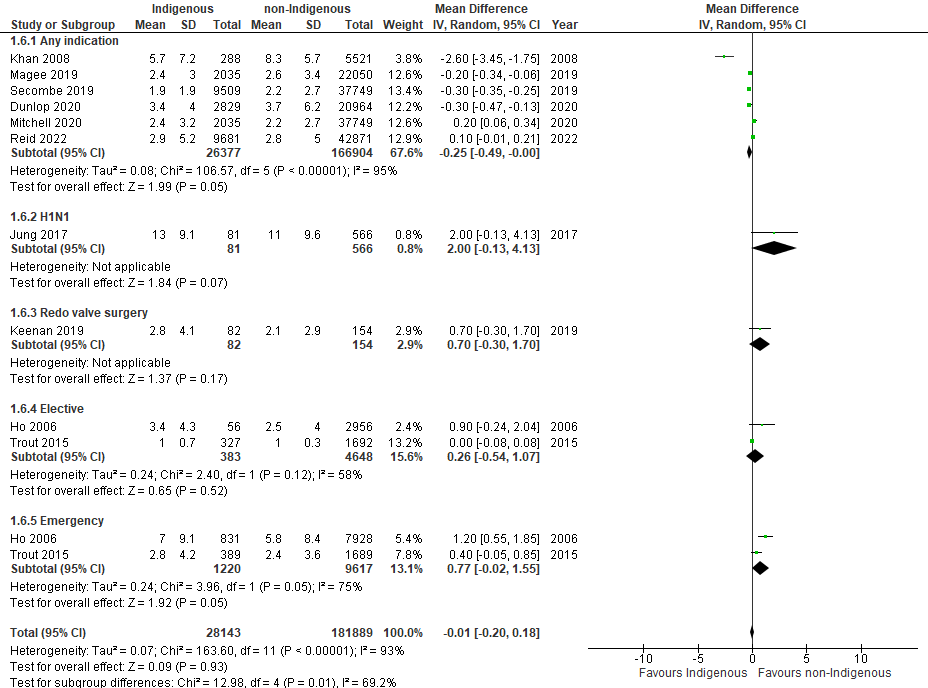


**eFigure 6C.** Forest plot of receipt of invasive mechanical ventilation: subgroup analysis by indication.


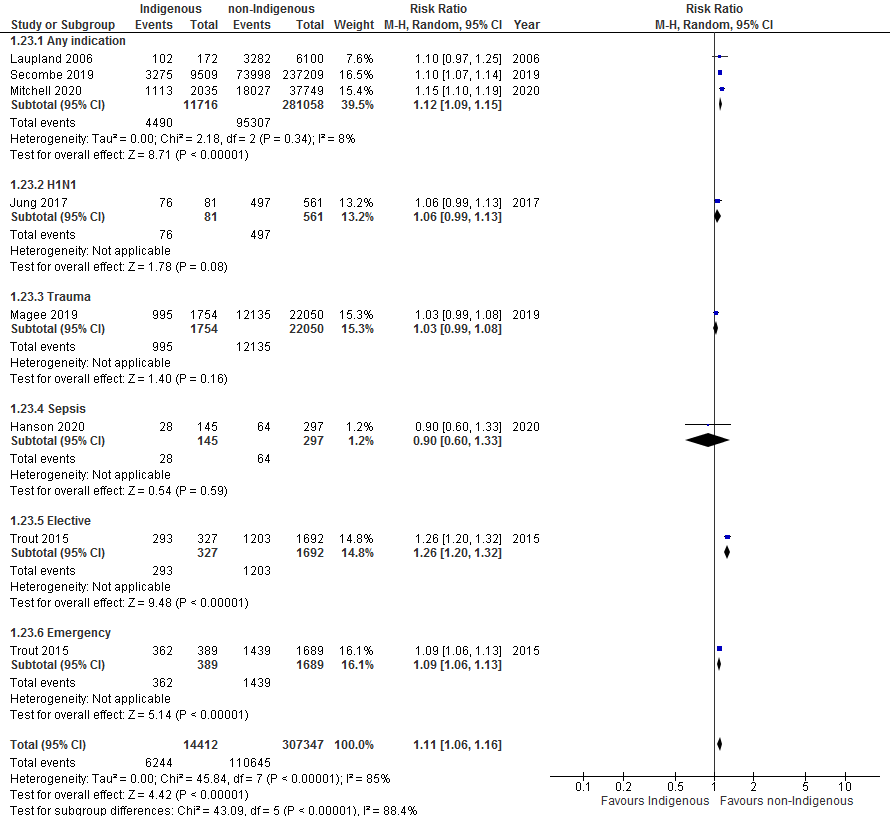


**Supplementary File 7:** Forest plots of Indigenous vs. non-Indigenous populations for outcomes among an obstetric critically ill population.

**eFigure 7A.** Forest plot for ICU mortality among an obstetric population


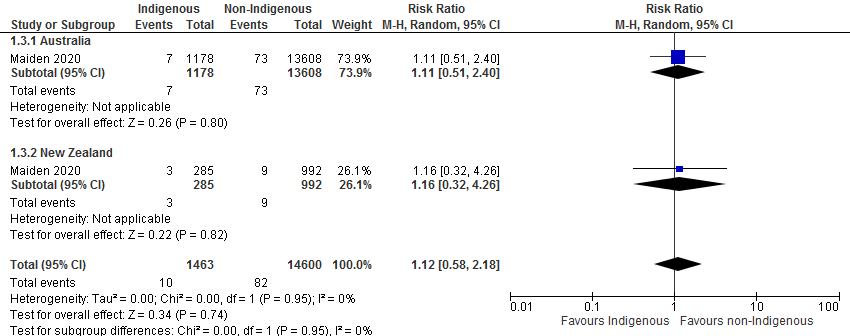


**eFigure 7B**. Forest plot for ICU length of stay among an obstetric population


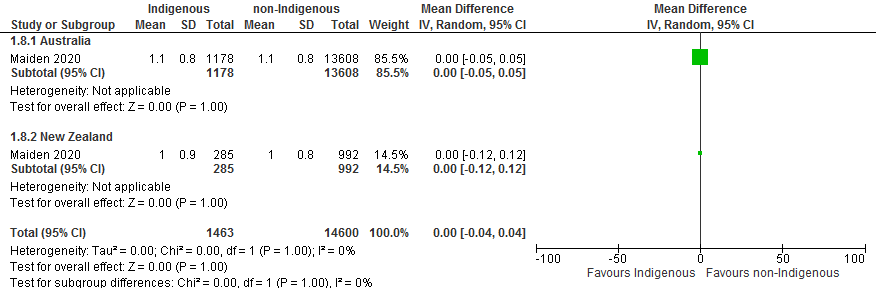


**eFigure 7C.** Forest plot for invasive mechanical ventilation among an obstetric population


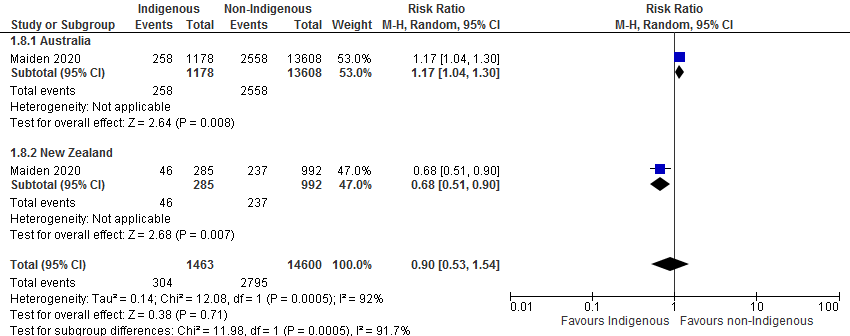


**Supplementary File 8:** Egger’s Funnel plot.


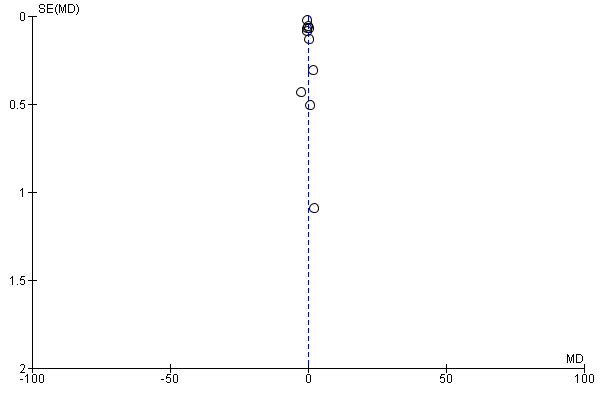

Supplement: Supplementary file 1 — Additional file 1: File S1. Full search strategy and search terms. File S2. Risk of bias assessments for included studies, using the Newcastle–Ottawa Scale. File S3. Summary of the Aboriginal and Torres Strait Islander Quality Assessment Tool appraisal. File S4. Ancillary analysis of outcomes not included in the main manuscript. File S5. GRADE evidence profile comparing Indigenous vs. non-Indigenous populations for the primary outcomes. File S6. Forest plots for of Indigenous vs. non-Indigenous for ICU mortality (Figure s6A), ICU Length of Stay (Figure s6B), and IMV (Figure s6C): subgroup analysis by indication. File S7. Forest plots of Indigenous vs. non-Indigenous populations among an obstetric critically ill population for: ICU mortality (Figure s7A), ICU LOS (Figure s7B), and IMV (Figure s7C). File S8. Egger’s Funnel plot for small study bias. [file 13054_2023_4570_MOESM1_ESM.docx]
